# 1373. Health-Related Quality of Life in Outpatient Adults With Medically Attended Acute Respiratory Infection

**DOI:** 10.1093/ofid/ofad500.1210

**Published:** 2023-11-27

**Authors:** Xueyan Zhang, MaryPatricia Nowalk, Theresa M Sax, Helen D’Agostino, G K Balasubramani

**Affiliations:** University of Pittsburgh, Pittsburgh, Pennsylvania; University of Pittsburgh, Pittsburgh, Pennsylvania; University Of PIttsburgh, Pittsburgh, Pennsylvania; University of Pittsburgh, Pittsburgh, Pennsylvania; University of Pittsburgh, Pittsburgh, Pennsylvania

## Abstract

**Background:**

The COVID-19 pandemic has caused a significant global burden. However, there is limited evidence on the physical and mental health-related quality of life after mild COVID-19 infection.

**Methods:**

A prospective cohort study was conducted among adults (18 - 87 years) with acute respiratory infection from 3/30/2020 to 4/30/2021. RT-PCR tests confirmed SARS-CoV-2 infections from nasopharyngeal or nasal swab specimens. A Short Form Health Survey (SF-12), which measures physical and mental health functioning in the last four weeks, was administered at presentation for treatment in outpatient settings (enrollment) and at 6-8 weeks follow-up. Participants were classified based on enrollment SF-12 item responses using K-means clustering. Chi-square tests and ANOVAs were used to examine demographic and health characteristics by cluster. Linear regression was used to evaluate predictors of change in physical and mental health composite scores (PCS and MCS, respectively) from enrollment to follow-up.

**Results:**

856 COVID-unvaccinated patients completed both surveys. 352 subjects were grouped in the high-functioning group (HFG) at enrollment, 362 were in the medium-functioning group (MFG), and 151 were in the low-functioning group (LFG). At enrollment, compared with HFG, LFG was more likely to be female, have a lower education level, have a history of smoking, and experience symptoms including fever, chills, shortness of breath, vomiting or nausea, diarrhea, and loss of smell or taste. Changes from enrollment to follow-up in PCS and MCS were compared across groups. Increases in PCS and MCS were significantly smaller (1.4 ± 6.0, -1.6 ± 8.1) in HFG than in the MFG (5.9 ± 8.3, 0.6 ± 10.2) and the LFG (7.1 ± 9.2, 1.9 ± 10.7; P < 0.001 for both). In regression analyses, predictors of improved PCS and MCS were having COVID-19 illness, reporting shortness of breath, and being in the LFG compared with the HFG (P < 0.05 for all).

Cluster difference identified by canonical discriminant analysis

**Figure:**
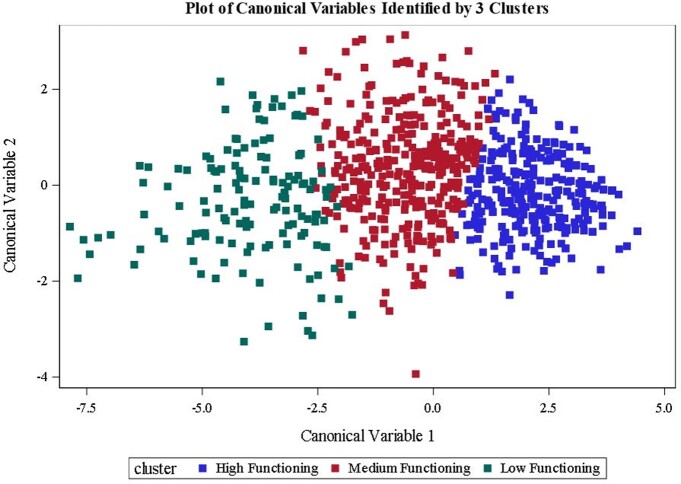

**Conclusion:**

Both physical and mental quality of life scores following an acute respiratory infection were better among those with mild COVID-19 illness than among those with non-COVID respiratory illnesses. Furthermore, changes in scores during follow-up were more resilient among those in the LFG, with smaller differences in scores across groups at follow-up than were evident at enrollment.

**Disclosures:**

**MaryPatricia Nowalk, PhD, RDN**, Merck & Co.: Grant/Research Support|Merck & Co.: Honoraria|Sanofi: Grant/Research Support

